# Adapting and testing a tool to map digital health resources use by older adults in Israel and Taiwan

**DOI:** 10.1093/eurpub/ckac129.157

**Published:** 2022-10-25

**Authors:** D Levin-Zamir, O Baron-Epel, P Chang, E Neter, E Eliahu, T Duong

**Affiliations:** 1 School of Public Health, Tel Aviv University, Tel Aviv, Israel; 2 School of Public Health, University of Haifa, Haifa, Israel; 3 Ruppin Academic Center, Emeq Hefer, Israel; 4 Show Chwan Memorial Hospital, Lugan, Taiwan; 5 School of Nutrition, Taipei Medical University, Taipei, Taiwan; 6 Department of Health Education and Promotion, Clalit, Tel Aviv, Israel

## Abstract

**Background:**

Despite the potential of digital health tools for improving health outcomes, older adults are known to use digital health tools differently than younger adults. Focusing on needs of older populations is critical, as their numbers and proportions are projected to increase dramatically in the coming decades, both in Israel and in Taiwan. A bi-national collaboration was developed to map existing digital health resources available to older adults, as part of a larger study on digital health services use among older adults.

**Methods:**

A mapping tool was adapted from the WHO classification of digital health interventions, based on the experience in the Taiwanese and Israeli health systems. The areas included public health, prevention, self- monitoring and self-care information and services in primary and tertiary care. The mapping documented digital resources offered by governmental/Ministry of Health, public primary care (HMOs), hospitals, and non-governmental organizations. Sources of information were institutional websites, evaluated by two specially trained reviewers for each organization who assigned a dichotomous value (yes/no) for each category. Interrater reliability was computed using a Kappa coefficient.

**Results:**

The instrument included 17 categories and 44 sub-categories of digital resources, ranging from public health information for emergency situations to specific health service characteristics. To date, the Kappa coefficients range from 0.59-0.68 for NGO, MOH and hospital resources, considered substantial; for 3 HMOs, the values ranged from 0.41-0.49, considered moderate.

**Conclusions:**

The mapping tool adapted to the countries’ digital resources allowed for bi-national research to compare/contrast the countries’ experience. The next stage of the study will validate the results through expert interviews, followed by an end user survey with older adults to assess both reported use of services and enabling digital health literacy skills.

**Key messages:**

• To meet the needs of aging populations, attention needs to be given to their engagement with digital health services and resources.

• Mapping digital health resources is essential for estimating how health needs are met nationally.

